# Green propolis extract replaces salinomycin in the high-concentrate diet of sheep

**DOI:** 10.1007/s11250-026-05012-x

**Published:** 2026-04-02

**Authors:** Natalia Ingrid Souto da Silva, Thiago Luís Alves Campos de Araújo, Salenilda Soares Firmino, Maria Mirna Gonçalves Oliveira, Emerson Marcos Nascimento Silva, Raiane Mikaeli Nunes da Costa, José Gustavo Lima de Almeida, Elisomar André da Silva, Stela Antas Urbano, Dorgival Morais de Lima Júnior

**Affiliations:** 1 Universidade Federal Rural do Semi-Árido, Francisco Mota Steert, 572, Mossoró, RN 59625-900 Brazil; 2 Universidade Federal do Rio Grande do Norte, Jundiaí District, Macaíba, RN 59280-000 Brazil

**Keywords:** *Mimosa tenuiflora*, Nitrogen metabolism, Phytogenic additive, Water intake

## Abstract

This study aimed to evaluate the effects of supplementing high-concentrate diets for sheep with green propolis extract (PRO) from *Mimosa tenuiflora* or with salinomycin (SAL) on feed intake, apparent digestibility, nitrogen balance, and water balance. Three sheep, ½ Dorper x ½ Santa Inês, were assigned to a 3 × 3 Latin Square design, repeated over time, totaling six replicates per treatment. The animals were castrated, with a mean age of 26 months and a weight of 47.94 ± 4.73 kg. The treatment diets were control (CON); CON + 24 mg SAL/kg DM (SAL); and CON + 24 mL PRO/day (PRO). The experiment lasted 102 days, each consisting of 17 days of adaptation and five days of data collection. Intake of dry matter (DM), organic matter (OM), crude protein (CP), neutral detergent fiber, and non-fibrous carbohydrates did not differ (*P* > 0.05) between the additives. However, total digestible nutrient (TDN) intake was lower (*P* < 0.05) for the SAL diet. Similarly, the SAL diet tended to reduce the apparent digestibility of DM (*P* = 0.0542), OM (*P* = 0.0573), CP (*P* = 0.0662), and TDN (*P* = 0.0836) in sheep. In addition, SAL-fed sheep absorbed and retained less (*P* < 0.05) nitrogen than those fed the CON and PRO diets. Supplementation with salinomycin at 24 mg/kg DM impairs energy intake and nitrogen utilization by sheep. Supplementation with 24 mL/day of green propolis extract does not affect energy intake and nitrogen use by sheep.

## Introduction

The reduction in the time required to finish sheep primarily results from the intensification of production systems. Increasing the energy density of diets is one of the most used strategies to achieve this goal (Silva et al. 2023). Therefore, understanding nutrient utilization by these animals is essential to increase production efficiency and optimize the use of natural resources (Lu 2024).

Increasing dietary energy, mainly through higher concentrate proportions, improves feed efficiency (Jiang et al. 2022) and productive performance by optimizing the use of metabolizable energy for tissue deposition (Parente et al. 2016). However, it should be considered that ruminants evolved to adapt to predominantly fibrous diets. High intake of concentrates can compromise rumen homeostasis, potentially leading to adverse effects on fermentation and animal health, including a higher incidence of ruminal acidosis, resulting in production losses and increased veterinary costs (Coon and Tucker 2024; Squizatti et al. 2023).

Given the high fermentative capacity of high-concentrate diets, the strategic inclusion of nutritional additives (ionophores, essential oils, propolis etc.) has proven effective in mitigating rumen acidification (Pacheco et al. 2023; Casagrande et al. 2024) and promoting microbial ecosystem stability (Dellaqua et al. 2024; Ovani et al. 2026). By preserving proper rumen function, these interventions contribute to more efficient nutrient utilization (Moura et al. 2021) and greater host metabolic resilience, thereby improving feed efficiency and animal performance (Squizatti et al. 2023).

Among the available alternatives, ionophore antibiotics remain the most widely used additives in intensive production systems (Monsalve and Millen 2025). However, although their efficacy is well recognized, the use of these compounds has been restricted in some countries due to concerns about antimicrobial resistance and its implications for public health (Aguiar et al. 2022). These issues have motivated regulatory restrictions in different consumer markets, which reinforces the need to identify and validate alternative nutritional strategies, such as plant extracts.

Green propolis, obtained from *Mimosa tenuiflora* shoots, is rich in bioactive compounds with antimicrobial and antioxidant properties (Cécere et al. 2021; Tiveron et al. 2016) can modulate fermentation and improve nutrient utilization, similarly to ionophores, but without the risks associated with microbial resistance and the presence of residues (Zawadzki et al. 2011). Recently, our group found evidence that supplementation with 24 mL/day of green propolis extract from *Mimosa tenuiflora* improves immune and liver function in sheep (Silva et al. 2025a).

Although studies indicate positive effects of propolis on rumen stability (Silva et al. 2021) and nutrient digestibility (Paixão et al. 2022), there are few studies comparatively evaluating the use of green propolis from *Mimosa tenuiflora* and ionophore additives on nutrient utilization by sheep. Thus, this study aimed to evaluate whether supplementing sheep diets with 24 mg salinomycin/kg dry matter or 24 mL/day of green propolis extract affects feed intake, digestibility, nitrogen balance, and water balance in sheep fed high-concentrate diets. The hypothesis was that both additives would improve nutrient utilization efficiency and water homeostasis in high-concentrate diets.

## Materials and methods

### Experimental period and location

The experiment was conducted at the Universidade Federal Rural do Semi-Árido (UFERSA), Mossoró, Rio Grande do Norte, Brazil, with the following geographical coordinates: Latitude: 5°11’17” South and Longitude: 37°20’39” West. The region’s climate is classified as BSwh’ by the Köppen classification. The average temperature during the experiment was 27.8 °C.

### Obtaining propolis and making the extract

The crude samples of green propolis from *Mimosa tenuiflora* were acquired from apiaries located in Limoeiro/CE. The samples were manually cleaned to remove impurities (such as shell fragments or dead insects), then frozen and crushed in a grain crusher. To obtain the extract, 30 g of ground propolis was stored in glass bottles, and 100 mL of 70% hydroalcoholic solution of GL was added. The resulting solution was left to rest for 12 days in a dark location, after which it was centrifuged. Then, the extract was stored in amber-colored glass bottles at refrigerated temperatures, as described by Stradiotti Júnior et al. (2004).

The hydroalcoholic extract of green propolis was subjected to phytochemical characterization to determine the antioxidant activity and the content of total phenols and total flavonoids. Antioxidant activity was evaluated using the DPPH free radical reduction method, as described by Velázquez et al. (2003). The total phenol content was determined by the Folin-Ciocalteau reagent, according to Meda et al. (2005). The results were extrapolated to a calibration curve prepared with a gallic acid standard (20 to 200 ppm) and expressed as grams gallic acid equivalent (GAE)/100 mL of extract. The total flavonoid content was determined according to Meda et al. (2005), with adaptations. The results were expressed in grams of quercetin equivalent (QE)/100 mL of extract, using a quercetin standard curve (5 to 50 mg/L) (Table 1).

**Table 1:** Total antioxidant, phenolic and flavonoid contents of hydroalcoholic extract of green propolis (*Mimosa tenuiflora*)

Contents	Mean ± SD
Total phenolic (g/100 g of GAE)	8.04 ± 0.3
Flavonoids (g/100 g of QE)	3.49 ± 0.4
Antioxidant activity (mg of DPPH)	34.4 ± 0.1

GAE = gallic acid equivalent; QE = quercetin equivalent; DPPH = 2,2-diphenyl-1-picrylhydrazyl neutralized per milliliter of extract

### Animals and treatments tested

We used three castrated male sheep, ½ Dorper x ½ Santa Inês, with a mean age of 26 months and a weight of 47.94 ± 4.73 kg. Before the experiment, the animals received treatments against endo- and ectoparasites (Ripercol^®^ L, Zoetis, USA) and were housed in metabolism cages, each provided with a feeder, drinker, and feces and urine collection trays. The metabolism cages were installed in a shed covered with clay tile, with a ceiling height of 3.5 m, a concrete floor, and artificial lighting at night.

The experimental design used was the Latin square (3 × 3) repeated over time, totaling six replicates per treatment. The experiment lasted 102 days and was divided into six periods of 17 days: 12 days for animal adaptation and five days for data collection and parameter evaluation. The treatment diets were: (1) control diet, without additives (CON); (2) CON + 24 mg salinomycin/kg DM (SAL); and (3) CON + 24 mL of hydroalcoholic extract of green propolis (*Mimosa tenuiflora*) (PRO), randomly distributed to the three animals in each period.

Diets were calculated to meet the weight gain of 200 g/day (NRC 2007). The forage: concentrate ratio of the diet was 20:80. The concentrate consisted of crushed corn grain, soybean meal, wheat bran, mineral core, calcitric limestone, and sea salt (NaCl) (Table 2). Tifton 85 hay was purchased in bales of 15–18 kg, crushed in a forage mill, adjusted to produce 30 mm particles, placed in plastic bags, and stored in a dry location.

**Table 2:** Participation of the ingredients and composition of the experimental diet of lambs fed high-concentrate diets and receiving green propolis extract or salinomycin

			CON	SAL	PRO
Tifton hay 85 (%)			20	20	20
Corn grain (%)			58.37	58.37	58.37
Wheat bran (%)			12.04	12.04	12.04
Soybean meal (%)			7.84	7.84	7.84
Sea salt (%)			0.72	0.72	0.72
Mineral core (%)*			0.72	0.72	0.72
Calcitic limestone (%)			0.36	0.36	0.36
Total			100	100	100
	Tifton 85 hay	Concentrate	Diet		
Dry matter (g/kg as fed)	93.44	92.29	92.52		
Ash (g/kg DM)	8.8	4.08	5.02		
Organic matter (g/kg DM)	91.2	95.92	94.98		
Ether extract (g/kg DM)	2.23	3.65	3.37		
Crude protein (g/kg DM)	11.99	13.47	13.17		
NDFap (g/kg DM)	69.08	12.30	23.66		
NFC (g/kg DM)	7.90	66.50	54.78		

^1^ 24 mg/kg DM/day; ^2^ 24 mL/day

*Guarantee levels (1.000 g): Calcium 120 g; Phosphorus 87 g; Sodium 147 g; Sulfur 18 g; Cobalt 40 mg; Copper 590 mg; Iodine 80 mg; Chromium 20 mg; Manganese 1300 mg; Selenium 15 mg; Zinc 3800 mg; Iron 1800 mg; Molybdenum 10 mg; Fluorine (max.) 870 mg. CON = Control; SAL = Salinomycin; PRO = Propolis; NDFap ^=^ Neutral detergent fiber corrected for ash and protein; NFC = Non-fibrous carbohydrates

The daily feed supplementation was divided into two meals, at 8:00 and 16:00, with total mixed feed. Voluntary intake was ensured by supplementing feed with 10% leftovers from the previous day. The administration of propolis extract was performed orally using a 50 cc automatic vaccinator with an oral dosing nozzle (Model 91, Walmur^®^, Brazil) to ensure daily supplementation of the recommended levels of PRO. The total dose of propolis extract was administered twice, with half given at 8:00 and the other half at 16:00, thirty minutes after offering the diet. Animals that did not receive propolis extract received 15 mL of water as a placebo solution. Salinomycin (brand) was mixed with the concentrate at a dose of 24 mg/kg dry matter. The feed was given as a total mixture twice daily, at will, to allow for 10% leftovers.

Samples of the supplemented diet, leftovers, feces, and urine were collected over the five-day data collection period to determine nutrient intake, apparent digestibility, and nitrogen balance.

### Nutrient intake and digestibility

The amounts of the diet supplemented, and leftovers were recorded daily to determine consumption. Thus, nutrient intake was determined by the difference in chemical composition between the feed provided and the collected leftovers. Samples of the supplemented feed and leftovers were collected, stored in plastic bags, and frozen at -15 °C until analysis. Nutrient apparent digestibility was determined using the total feces collection method. The feces produced by the animals were collected in plastic trays adapted to the metabolism cage. The contents of the feces collection trays were weighed every 24 h, always at 07:00, followed by homogenization and sampling of 10% of the total excreted feces weight (Rymer 2000). The samples were sprayed with sulfuric acid solution (1:1), placed in plastic bags, and stored at -15 °C until analysis.

### Chemical composition analysis

Samples of feed, leftovers, and feces were thawed for 24 h in a refrigerator (4 °C), homogenized, and pre-dried in a forced-ventilation oven (Model TE-394/2-MP, Tecnal^®^, Brazil) at 55 °C for 72 h. After pre-drying, the material was ground in a knife mill (Model R-TE-680, Tecnal^®^, Brazil) with a 1 mm sieve and placed in hermetically sealed containers for further analysis of the chemical composition.

The samples were analyzed for their chemical constituents using the methods described in (AOAC, 2019) for dry matter (DM) (Method no. 934.01), ash (MM) (Method no. 942.05), crude protein (CP) (Method no. 981.10), ether extract (EE) (Method no. 920.39), and neutral detergent fiber corrected for ash and protein (mm) (Method no. NDFap), as described by Van Soest et al. (1991) with adaptations by Licitra et al. (1996); and Mertens (2002) for ash and protein correction, respectively. Non-fibrous carbohydrates (NFC) were calculated as described by Detmann and Valadares Filho (2010). The total digestible nutrient (TDN) content of the diets was estimated using the equation described by Weiss et al. (1992).

### Nitrogen balance

Urine was collected in plastic trays attached to the metabolism throughout the collection period. To prevent nitrogen evaporation, 20 mL of sulfuric acid (1:1) was added to the trays. All urine was weighed daily, and a 10% sample of the total volume was collected, packaged in bottles, and frozen at -10 °C until analysis. A urine aliquot was used to determine total nitrogen (AOAC, 2019; method no. 981.10). The nitrogen absorbed (g/day) was determined by the difference between the nitrogen ingested and excreted in the feces. Retained nitrogen was determined by consumed nitrogen (g/day) - (nitrogen excreted in feces (g/day) + nitrogen excreted in urine (g/day).

### Water balance

Total water intake was determined during the five-day collection period for each, quantified through both drinking and feed intake. Water was supplemented in 15-liter polyethylene buckets placed in the metabolism cage. Water intake was determined by daily weighing, recording the difference between the weight of the water provided and the weight of the remaining water. Water losses through evaporation were determined by weighing two buckets of water strategically placed in the facility. Evaporation data were used to correct water intake. Dietary water intake was estimated by determining the moisture content of feed and leftovers. The total water excreted was determined by the sum of the volumes of water excreted in feces and urine.

### Statistical analysis

Statistical analyses were performed using the SAS on Demand for Academics platform. Assumptions of normality and variance homogeneity were evaluated using the Shapiro-Wilk and Levene tests, respectively. The data were submitted to the analysis of variance using the PROC MIXED procedure, considering the following model:

$$Yijk\, = \,\mu \, + \,Ti\, + \,Qj\, + \,Pk + \left( {A/Q} \right)lj\, + \,T*Qij\, + \,\varepsilon ijk$$

Where: Yijk = observation ijk; µ = over mean; Ti = fixed effect of treatment i; Qj = fixed effect of square j; Pk = random effect of period k; (A/Q)lj = random effect of animal l into square j; T*Qij = fixed effect of treatment i and square j interaction; εijk = random residual error. The Tukey-Kramer multiple-comparison test was applied when treatment effects were significant. A 5% probability level was adopted for all procedures.

## Results

The green propolis extract used presented concentrations of 8.04 g/100 g of total phenols, expressed as the equivalent of gallic acid, and 3.49 g/100 g of flavonoids, expressed as the equivalent of quercetin. Thus, the daily intake by the animals corresponded to 1.93 g of total phenols or 0.84 g of flavonoids in the PRO treatment. Additionally, the antioxidant activity of the propolis ethanolic extract was determined as the amount of neutralized DPPH per milliliter of extract. From this relationship, it was estimated that 1 mL of propolis ethanolic extract (300 mg/mL) could neutralize approximately 34.3 mg of DPPH, confirming its high antioxidant activity.

Supplementation with SAL and PRO showed no differences (*P* > 0.05) for the intake of dry matter, organic matter, ether extract, crude protein, neutral detergent fiber, and non-fibrous carbohydrates (Table 3). However, sheep from the SAL group consumed less total digestible nutrients (*P* = 0.0425) than animals from treatments CON and PRO.

**Table 3:** Nutrient intake and digestibility of the diet of sheep fed high-concentrate diets and receiving green propolis extract or salinomycin

	Treatments			SEM	*P*-value^*^
	CON	SAL	PRO		
Intake (kg/day)					
Dry matter	1.23	0.97	1.15	0.063	0.3094
Organic matter	1.17	0.93	1.09	0.060	0.3003
Mineral matter	0.06	0.05	0.05	0.004	0.3759
Ether extract	0.04	0.04	0.04	0.002	0.3589
Crude protein	0.16	0.13	0.16	0.007	0.3421
Neutral detergent fiber	0.28	0.20	0.23	0.023	0.2380
Non-fibrous carbohydrates	0.69	0.56	0.66	0.031	0.3008
Total digestible nutrients	0.97a	0.68b	0.99a	0.066	0.0425*
Apparent digestibility (%)					
Dry matter	77.26	66.58	80.45	2.650	0.0542
Organic matter	78.80	69.00	81.50	0.024	0.0573
Ether extract	79.38	72.84	79.13	1.816	0.2192
Crude protein	71.27	60.19	74.14	2.984	0.0662
Neutral detergent fiber	64.32	43.39	63.03	5.036	0.1941
Non-fibrous carbohydrates	85.26	82.37	89.17	1.600	0.1185
Total digestible nutrients	77.17	66.67	79.50	0.029	0.0836

CON = Control; SAL = Salinomycin; PRO = Propolis; SEM = Standard error of the mean; ^*^Treatments with statistical differences are given distinct letters

Regarding apparent digestibility, there was a trend in the effect of additive supplementation on the apparent digestibility of dry matter (*P* = 0.0542), organic matter (*P* = 0.0573), and crude protein (*P* = 0.0662), with lower digestibility in the SAL group.

Nitrogen intake did not differ between treatments (*P* > 0.05), ranging from 21.6 to 26.8 g/day. Similarly, no differences (*P* > 0.05) were observed in fecal, urinary, or total nitrogen excretions. However, SAL-fed lambs showed lower (*P* = 0.0387) nitrogen uptake (16.8 g/day) compared to the CON (19.1 g/Day) and PRO (19.4 g/day) groups (Table 4).

**Table 4:** Nitrogen balance of lambs fed high-concentrate diets and receiving green propolis extract or salinomycin

	Treatments			SEM	*P*-value^*^
	CON	SAL	PRO		
Nitrogen intake (g/day)	26.8	21.6	26.2	1.250	0.1172
Excretion of fecal N (g/day)	7.77	7.97	6.77	0.563	0.6054
Urinary N excretion (g/day)	3.48	3.77	3.29	0.615	0.8890
Total N excretion (g/day)	11.3	11.7	10.0	0.637	0.3538
Nitrogen absorbed (g/day)	19.1a	13.6b	19.4a	1.232	0.0387*
N balance (%)	15.6	11.2	16.1	1.411	0.1016
Retained nitrogen (% absorbed)	15.58a	9.87b	16.12a	1.592	0.0258*
Retained nitrogen (% ingested)	57.1a	39.5b	59.8a	5.046	0.0167*

CON = Control; SAL = Salinomycin; PRO = Propolis; SEM = Standard error of the mean; ^*^Treatments with statistical differences are given distinct letters

Retained nitrogen, expressed as a percentage of absorbed nitrogen, was also lower (*P* = 0.0258) in the SAL group (9.87%) than in the CON (15.58%) and PRO (16.12%) groups. The same pattern was observed for retained nitrogen relative to ingested nitrogen, with higher values in the control (57.1%) and propolis (59.8%) groups compared to the salinomycin (39.5%) group (*P* = 0.0167).

Water intake ranged from 3.9 to 4.1 kg/day, with no differences (*P* > 0.05) among the CON, SAL, and PRO groups (Table 5). Likewise, the excretion of water in feces and urine, and the total excretion of water by sheep, did not differ between treatments (*P* > 0.05).

**Table 5:** Water balance of lambs fed high-concentrate diets and receiving green propolis extract or salinomycin

	Treatments			SEM	*P*-value^*^
	CON	SAL	PRO		
Water intake (kg/day)	3.90	4.16	4.00	0.2307	0.4945
Excretion of water via feces (g/day)	0.53	0.49	0.39	0.0471	0.3614
Excretion of water via urine (g/day)	0.91	1.12	0.93	0.1404	0.3040
Total water excretion (g/day)	1.44	1.61	1.32	0.1744	0.3484
Water absorbed (g/day)	3.37	3.67	3.61	0.2105	0.2747
Retained water (g/day)	2.46	2.55	2.68	0.1309	0.6309
Water retained (% of ingested)	63.6	63.6	68.8	2.7440	0.2703

CON = Control; SAL = Salinomycin; PRO = Propolis; SEM = Standard error of the mean; ^*^Treatments with statistical differences are given distinct letters

The values of water absorbed, and water retained were also similar between the groups (*P* = 0.2747 and *P* = 0.6309, respectively), as was the percentage of water retained relative to ingested, which ranged from 63.6% (control and salinomycin) to 68.8% (propolis), without statistical difference (*P* = 0.2703).

## Discussion

Dry and organic matter intake was similar, possibly due to the treatment feeds having identical percentages and chemical composition. This suggests that both salinomycin and green propolis extract can be used without a depressing effect on nutrient intake, an aspect often reported as limiting for some additives (Duffield et al. 2008). Nevertheless, the supplementation of ionophores can reduce DM intake in ruminants (Ahvanooei et al. 2023; Ribeiro et al. 2025) and even compromise animal energy intake. In this context, the lower total digestible nutrient intake observed for SAL-fed sheep compared to CON and PRO animals may be associated with the depression effects of DM intake linked to the lower digestibility of dry matter from the SAL group diet. When evaluating different doses of monensin sodium (0, 9,18, 27, and 36 ppm/DM) in the diet of Nellore steers, Pereira et al. (2019) observed that the increase in the inclusion of ionophore in diets caused a progressive reduction in dry matter intake.

The trend of lower apparent digestibility of dry matter and organic matter observed in SAL-fed sheep can be attributed to the change in the digestibility of crude protein in the entire digestive tract of the sheep (Chishti et al. 2021). Ionophores selectively alter microbial populations in the rumen, reducing degradation and fermentation processes in the rumen compartment (Marques and Cooke 2021). This can reduce the rate of bacterial growth and multiplication, influencing the fermentative dynamics of potentially degradable organic matter in the rumen (Azzaz et al. 2015; Marques and Cooke 2021) and, in turn, the degradation rates of ruminal DM and OM. Moharrery et al. (2014) reported that the degradation rates of ruminal DM have a high and positive correlation with the digestibility of DM in the entire digestive tract of ruminants.

In a recent study, Silva et al. (2025a) tested up to 24 mL/day of green propolis extract from *Mimosa tenuiflora*. They observed no effects of the additive on the apparent digestibility of nutrients. Similar results were reported by Silva et al. (2015) when comparing propolis extract with monensin in sheep diets, also without observing changes in digestibility. It is essential to note that in this study, the forage: concentrate ratio was 50:50, which may have influenced the animals’ response. Other factors such as propolis dosage, phenolic compounds level, or duration of administration may explain the lack of effect on nutrient digestibility in sheep diets (Silva et al. 2025b).

Diets with a higher starch content tend to increase the abundance of specific amylolytic bacteria, such as *Prevotella* spp. and *Selenomonas ruminantium*, without increasing microbial diversity, and, in some cases, even reducing it. This pattern results from the dominance of groups adapted to rapidly fermentable carbohydrates and tolerant of lower ruminal pH values. The modulation promoted by salinomycin, characterized by selective inhibition of gram-positive bacteria and favoring gram-negative bacteria, can strengthen this effect, directing fermentation toward greater propionate production and altering the balance between microbial groups. This scenario could partially explain the trend of reduction in apparent DM digestibility observed in the present study, even without significant changes in NDF digestibility.

Dry matter digestibility provides an overview of the efficiency of feed breakdown in the digestive tract but does not fully capture how the body harnesses the nitrogen ingested. To fully understand nutritional efficiency, it is also necessary to evaluate the metabolic fate of nitrogen —whether it is effectively absorbed and retained or lost, particularly in urine and feces. In the present study, lambs supplemented with salinomycin had lower nitrogen absorption and retention compared to the control and propolis groups. This is unexpected given the literature, which generally shows that ionophores do not compromise protein utilization; on the contrary, they are often associated with optimizing nitrogen metabolism in ruminants (Wanapat et al. 2024; Yang and Russell 1993).

Ionophores can improve the efficiency of protein utilization by three primary mechanisms: (i) diversion of ruminal fermentation to propionate, increasing energy efficiency and favoring the incorporation of nitrogen in protein synthesis; (ii) selective inhibition of proteolytic bacteria, which reduces excessive degradation of dietary protein and increases the fraction of *bypass* protein absorbable in the small intestine; and (iii) stabilization of the ruminal environment, which optimizes microbial protein (MICP) synthesis, ensuring a high-quality protein source for the animal (Dellaqua et al. 2024). However, the specific composition of the diet (prevalence of hard endosperm corn) may have interacted with salinomycin and influenced the fermentation rates of organic matter and ruminal crude protein, impairing the ruminal degradability of corn by selective inhibition of specific microbial groups (Palmonari et al. 2024; Plaizier et al. 2018). Subsequently, the undegraded corn from the rumen was not completely digested in the small intestine and was partially recovered in the feces. Although the literature indicates generally positive effects of ionophores on nitrogen utilization (Calsamiglia et al. 2010; Detmann et al. 2014), the results of the present experiment suggest that other factors, possibly related to the specific composition of the diet or the interaction with the individual microbiota of the animals, may have contributed to the reduction in nitrogen retention by the animals of the salinomycin group.

In this study, no significant differences in water intake were observed between the control, green propolis, and salinomycin groups. Similar results were obtained in previous work by our team, in which different levels of green propolis extract (*Mimosa tenuiflora*) did not affect the water balance of confined lambs (Silva et al. 2025a). These findings corroborate those of Ítavo et al. (2011), who also observed no effect of green propolis on water intake. On the other hand, Varela et al. (2023) reported a reduction in water demand in cows raised in a semi-arid environment when the dose of propolis extract increased from 32 mL/day to 64 mL/day. This divergence may be due to differences in species, environmental conditions, and supplementation protocols, suggesting that the effects of phytogenic on water intake remain incompletely elucidated.

## Conclusion

Sheep fed salinomycin consumed less energy and decreased utilization of dietary protein. Therefore, the use of 24 mg/kg of salinomycin in the high-concentrate diet for sheep is not recommended.

Green propolis extract does not detract from the nutritional value of the high-concentrate diet for sheep. Therefore, supplementation with 24 mL/day of green propolis extract from *Mimosa tenuiflora* can be considered, provided the economic context is favorable.

Both additives have been shown to benefit sheep metabolism. However, the green propolis extract from *Mimosa tenuiflora* stood out to favor greater nitrogen retention, suggesting additional potential for protein utilization in the diet.

## Data Availability

Not applicable.
